# Supplementary figures and images for: Polarised cell intercalation during Drosophila axis extension is robust to an orthogonal pull by the invaginating mesoderm
Source: PLoS Biol. 2024 Apr 29;22(4):e3002611. doi: 10.1371/journal.pbio.3002611 (PMC11081494; doi:10.1371/journal.pbio.3002611)

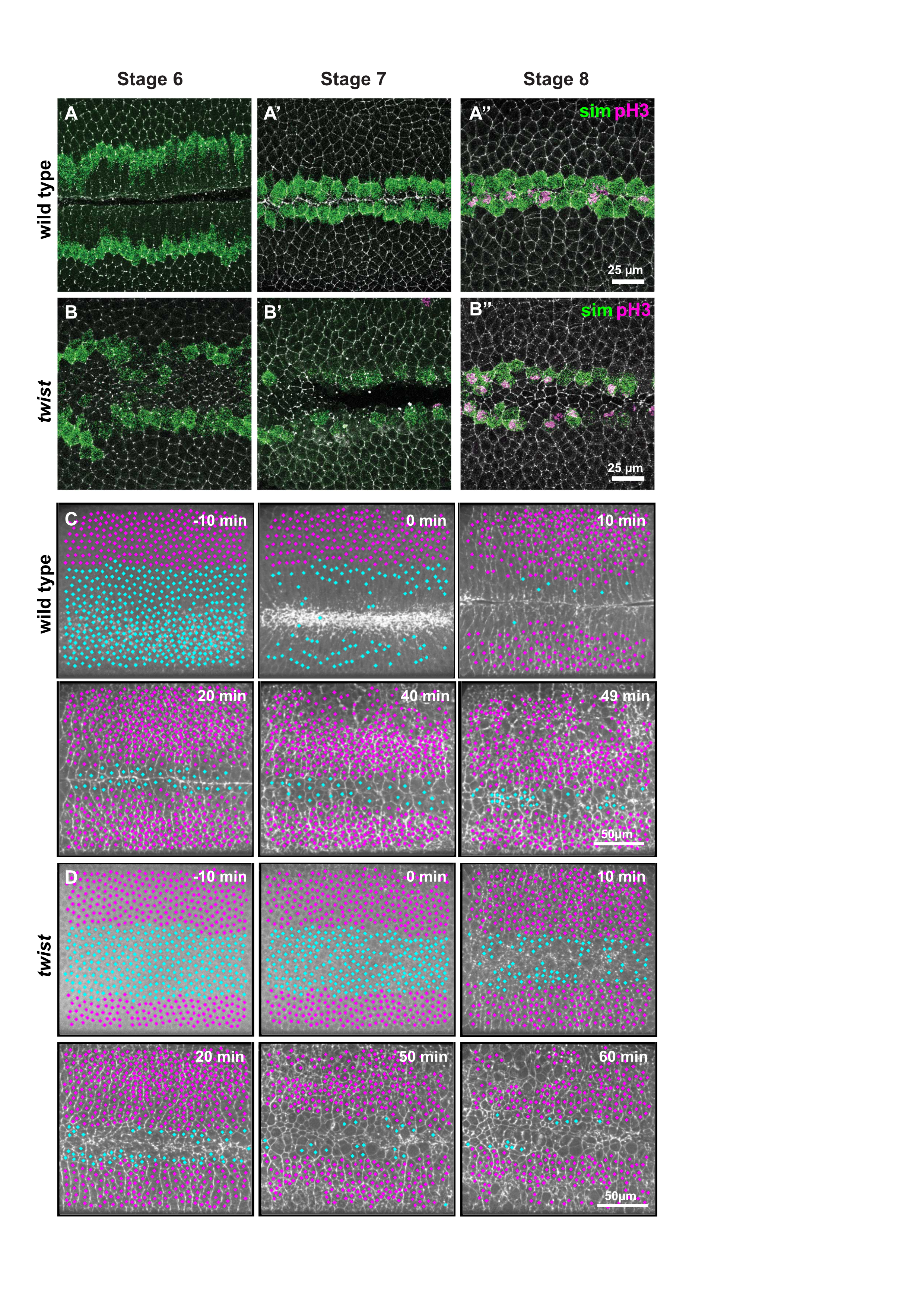

Supplement: S1 Fig — (A-B”) Representative images of in situ HCR of sim (green, mesectoderm) with antibody staining of pH3 (magenta, dividing cells) in stage 6 to 8 fixed embryos for wild type (A-A”) and twist (B-B”), used to inform definition of cell types in movies. (C, D) Examples of defining cell types in wild-type (C) and twist (D) movies at representative time points (mesoderm/mesectoderm, cyan; ectodermal germband, magenta), overlayed on Myosin II channel (before normalisation, maximum intensity projection). Unmarked cells are poorly tracked and excluded from the analysis. See also S3 and S4 Movies. HCR, hybridisation chain reaction; pH3, phosphoHistone3; sim, single-minded. (TIFF) [file pbio.3002611.s001.tiff]

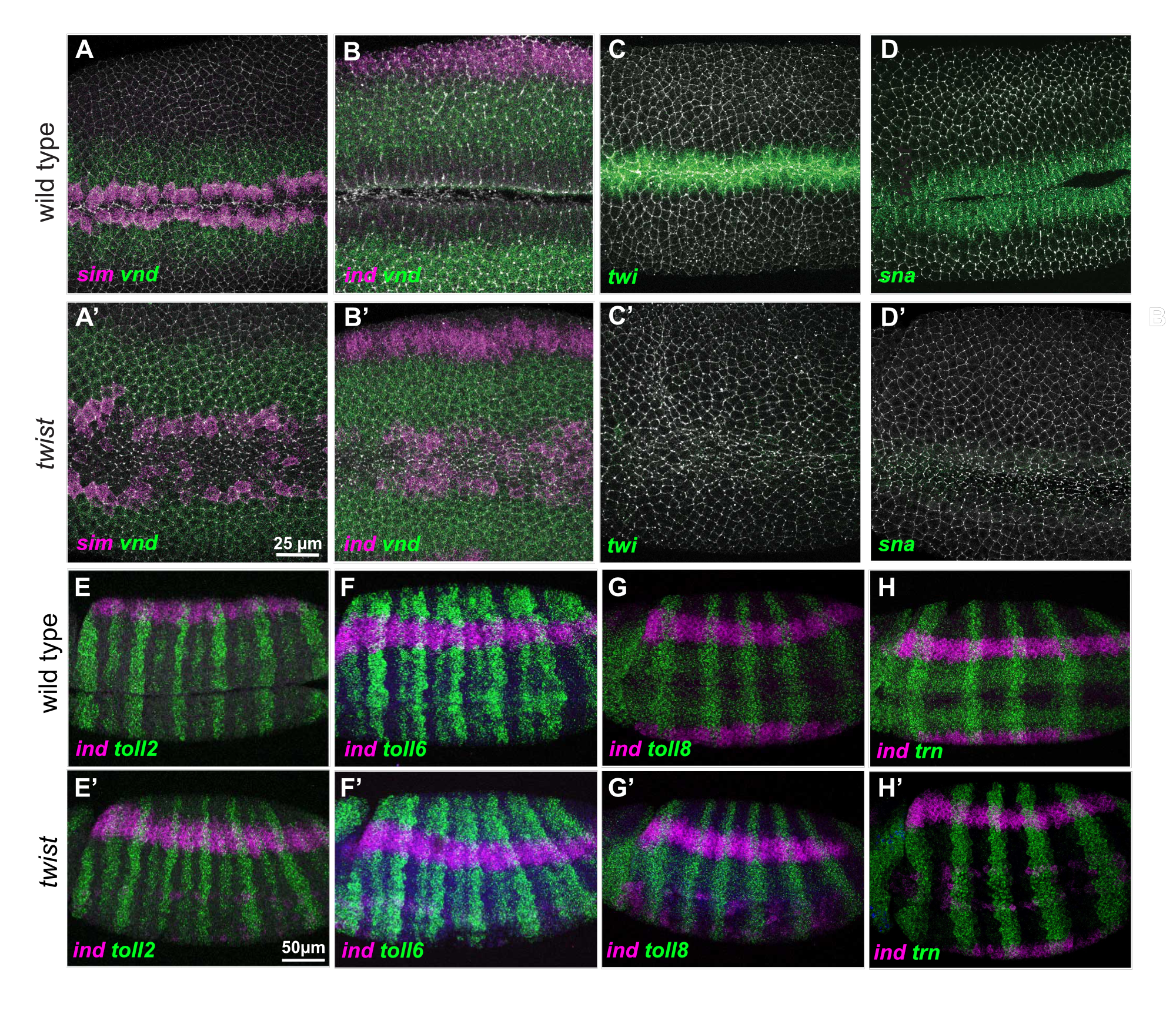

Supplement: S2 Fig — (A-D) In situ HCR of DV patterning genes in wild-type and twist mutant (A’-D’) stage 7 embryos: (A and A’) sim (magenta) and vnd (green); (B and B’) ind (magenta) and vnd (green); (C and C’) twist (green), which is missing in twist mutants (D and D’) snail (green), which is reduced and patchy in twist mutants as expected. (A-D’) are also marked with anti-pTyr (white, cell membranes). HCR of LRR receptors (green) in wild-type (E-H) and twist mutant (E’-H’) stage 7 embryos: (E and E’) expression of Toll-2; (F and F’) expression of Toll-6; (G and G’) expression of Toll-8; (H and H’) expression of trn. Maximum intensity projections of approximately 20 μm image stacks. (E-H’) are also stained for ind expression (HCR, magenta) and marked with pH3 (blue, dividing cells). DV, dorso-ventral; HCR, hybridisation chain reaction; ind, intermediate neuroblast defective; LRR, Leucine-rich repeat; pH3, phosphoHistone3; pTyr, phosphoTyrosine; sim, single-minded; trn, tartan; vnd, ventral nervous system defective. (TIFF) [file pbio.3002611.s002.tiff]

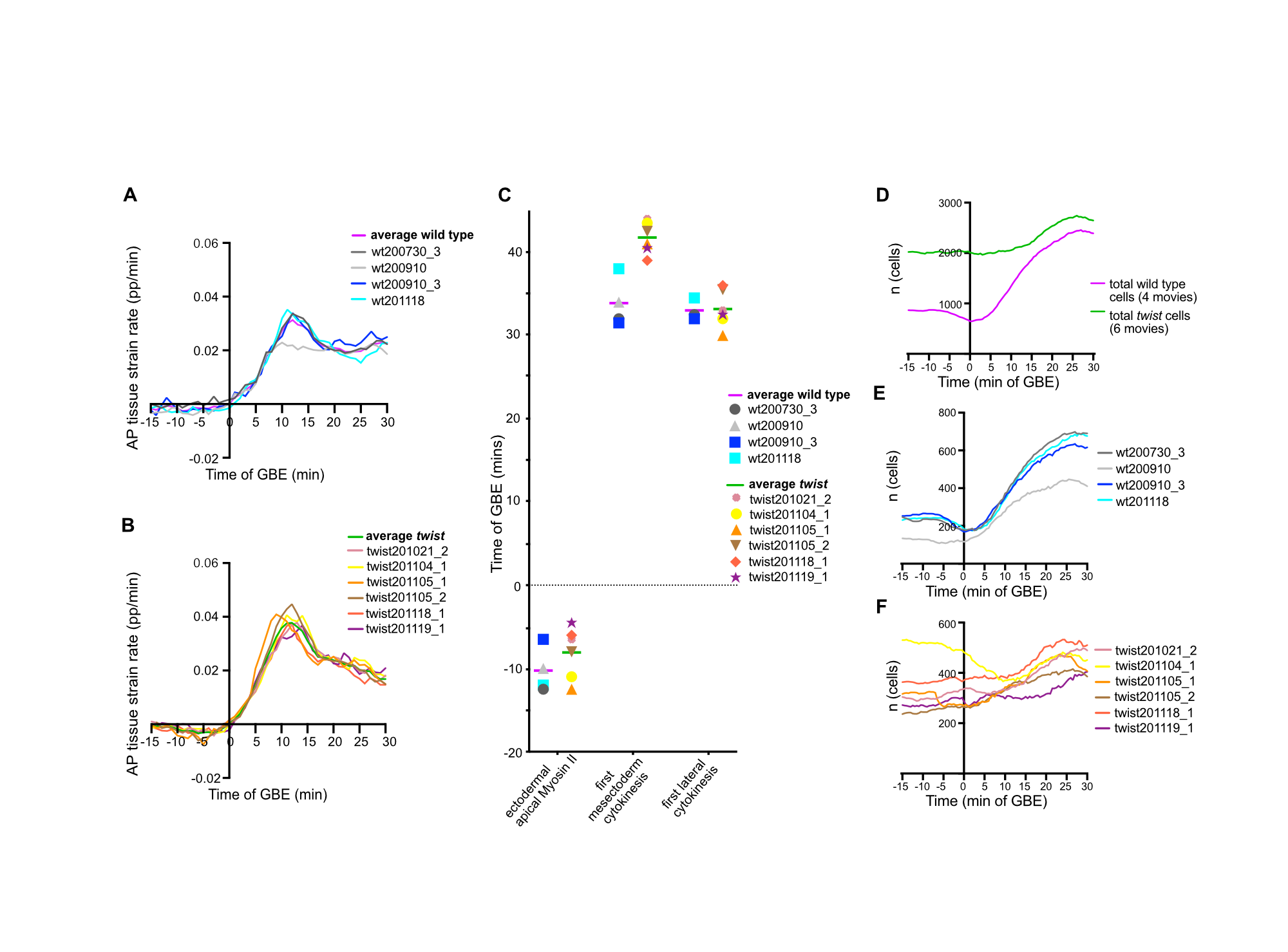

Supplement: S3 Fig — (A, B) Synchronisation of movies to start of GBE using tissue strain rates (proportion/minute), projected along AP axis in wild type (A) and twist (B). (C) Movie synchronisation was checked by mapping by eye using key developmental events visible in our field of view. Events in the ectoderm (“ectodermal apical Myosin II” and “first lateral cytokinesis” are clustered together within same genotype and between genotypes, showing that movie synchronisation is good throughout our window of study (−15 to +30 minutes of GBE). Note that the movies are longer than the −15 to 30 minutes of GBE analysed and vary slightly in length. Also, data are missing for “first lateral cytokinesis” for wt200910, due to the movie finishing too early for this to be assessed. Lines show average time of developmental events (magenta, wild type; green, twist). (D-F) Number of ectodermal cells analysed in wild-type and twist mutant movies over time. (D) Total number of cells analysed in all movies; (E) number of ectodermal cells analysed per wild-type movie over time; and (F) number of ectodermal cells analysed per twist movie over time. Data associated with this figure can be found in S6 Data. AP, antero-posterior; GBE, germband extension. (TIFF) [file pbio.3002611.s003.tiff]

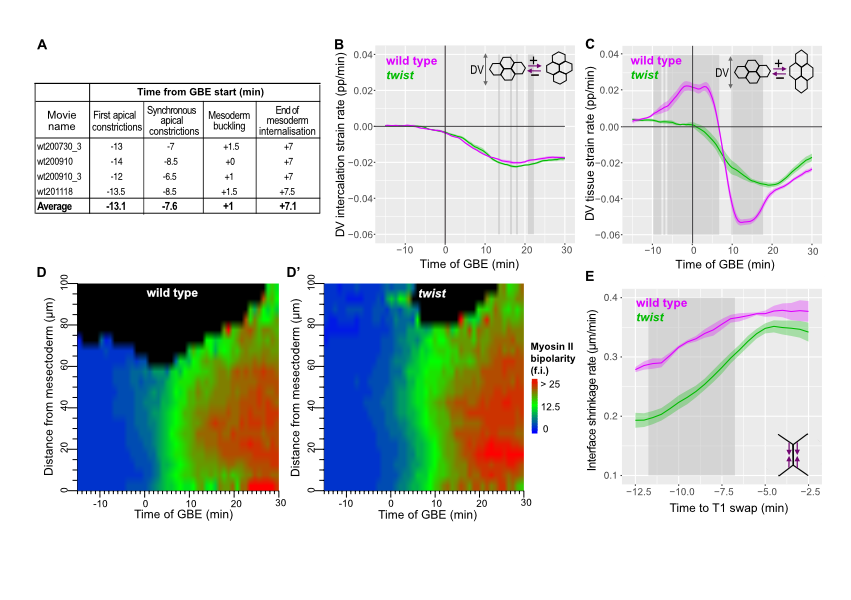

Supplement: S4 Fig — (A) Assessment by eye of timings of 4 events during mesoderm invagination in wild-type movies: when the first sustained apical constrictions begin; when synchronous apical constrictions along the AP axis become evident; when the central mesoderm has buckled and is surrounded by 2–3 rows of stretched mesoderm and mesectoderm cells; and when mesoderm cells are all internalised, with the mesectoderm cell row still stretched. Times show minutes of GBE. Averages of first and last events (about −13 and + 7 minutes) were used to draw “mesoderm invagination” bars in Fig 2C and 2E. (B) DV-projected intercalation strain rate of all tracked ectodermal cells, summarised for wild type (magenta) and twist (green) over time of GBE. Note that positive values would show intercalation contributing to extension in DV. Negative values show convergence in DV, with more negative values showing stronger convergence. For completion, we are also showing in (C), the DV-projected tissue strain rate of all tracked ectodermal cells, summarised for wild type (magenta) and twist (green) over time of GBE. Note that intercalation strain rate is calculated as tissue strain rate minus cell shape strain rate [40] (see S6B Fig). Therefore, DV tissue strain rate in C is very similar to DV cell shape strain rate (Fig 2C) before the start of GBE as DV cell intercalation strain rates are close to zero between −15 and 0 minutes of GBE. (D and D’) Spatiotemporal plots of unprojected Myosin II bipolarity of all tracked ectodermal cells, summarised for wild type (D) and twist (D’) against time of GBE and distance from mesectoderm (μm) (see Methods). (E) Speed of interface shrinkage (μm/minute) plotted against time to T1 swap during GBE (data for 0–30 minutes of GBE) summarised for wild type (magenta) and twist (green). Data associated with this figure can be found in S7 Data. AP, antero-posterior; DV, dorso-ventral; GBE, germband extension. (TIFF) [file pbio.3002611.s004.tiff]

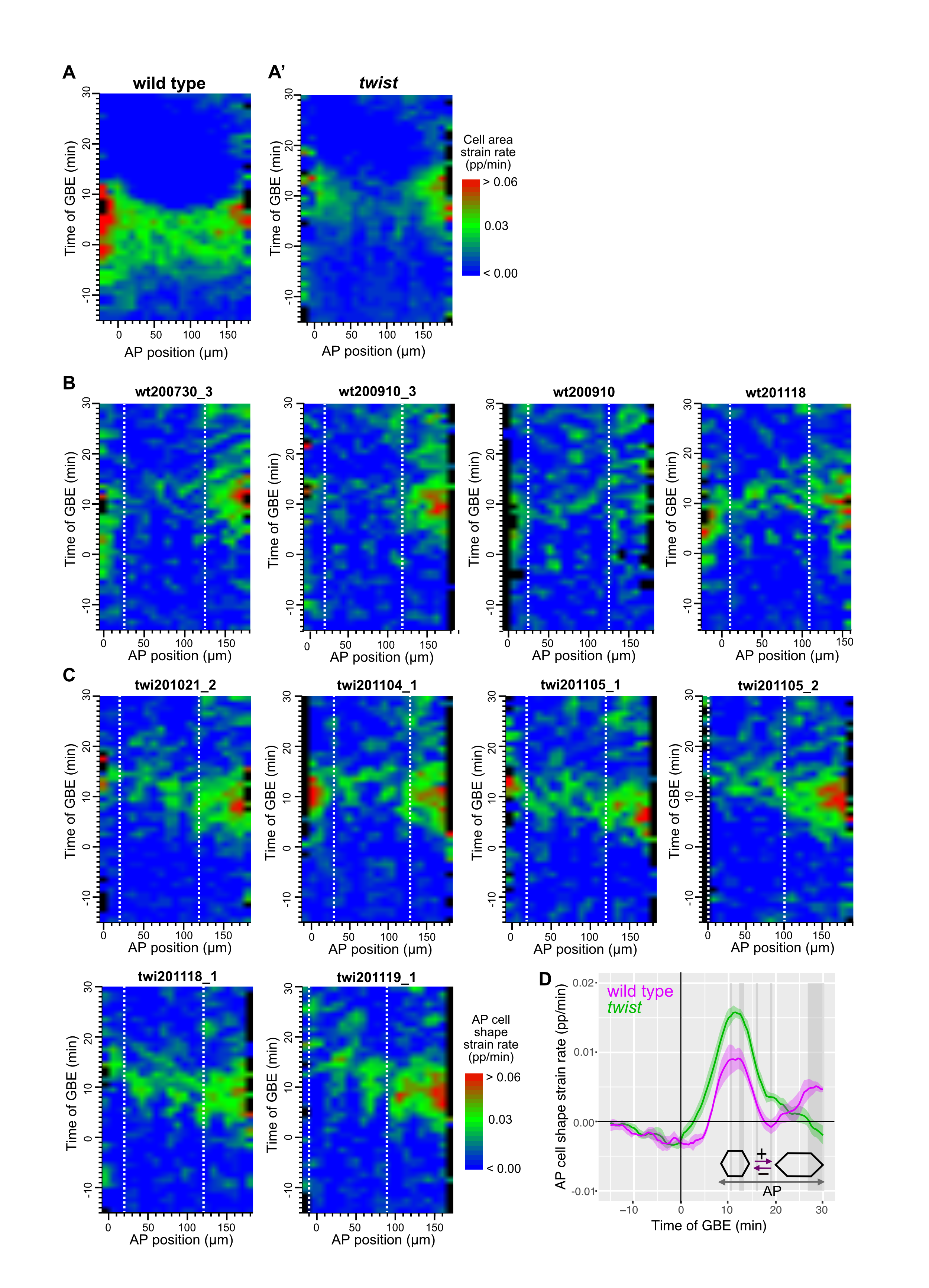

Supplement: S5 Fig — (A, A’) Spatiotemporal plots of cell area strain rate plotted against anterior-posterior position and time of GBE summarising 4 wild-type (A) and 6 twist (A’) movies. (B) Spatiotemporal plots of AP cell shape strain rate plotted against AP position and time of GBE for each wild-type movie. (C) Spatiotemporal plots of AP cell shape strain rate plotted against AP position and time of GBE for each twist movie. Vertical dotted white lines on each plot in (C) and (D) show the anterior and posterior limits of the 100 μm-wide central region used to plot data in D (see Methods). (D) AP cell shape change strain rate plotted against time of GBE summarising data from the central region shown in (B) and (C) for wild-type and twist movies. Data associated with this figure can be found in S8 Data. AP, antero-posterior; GBE, germband extension. (TIFF) [file pbio.3002611.s005.tiff]

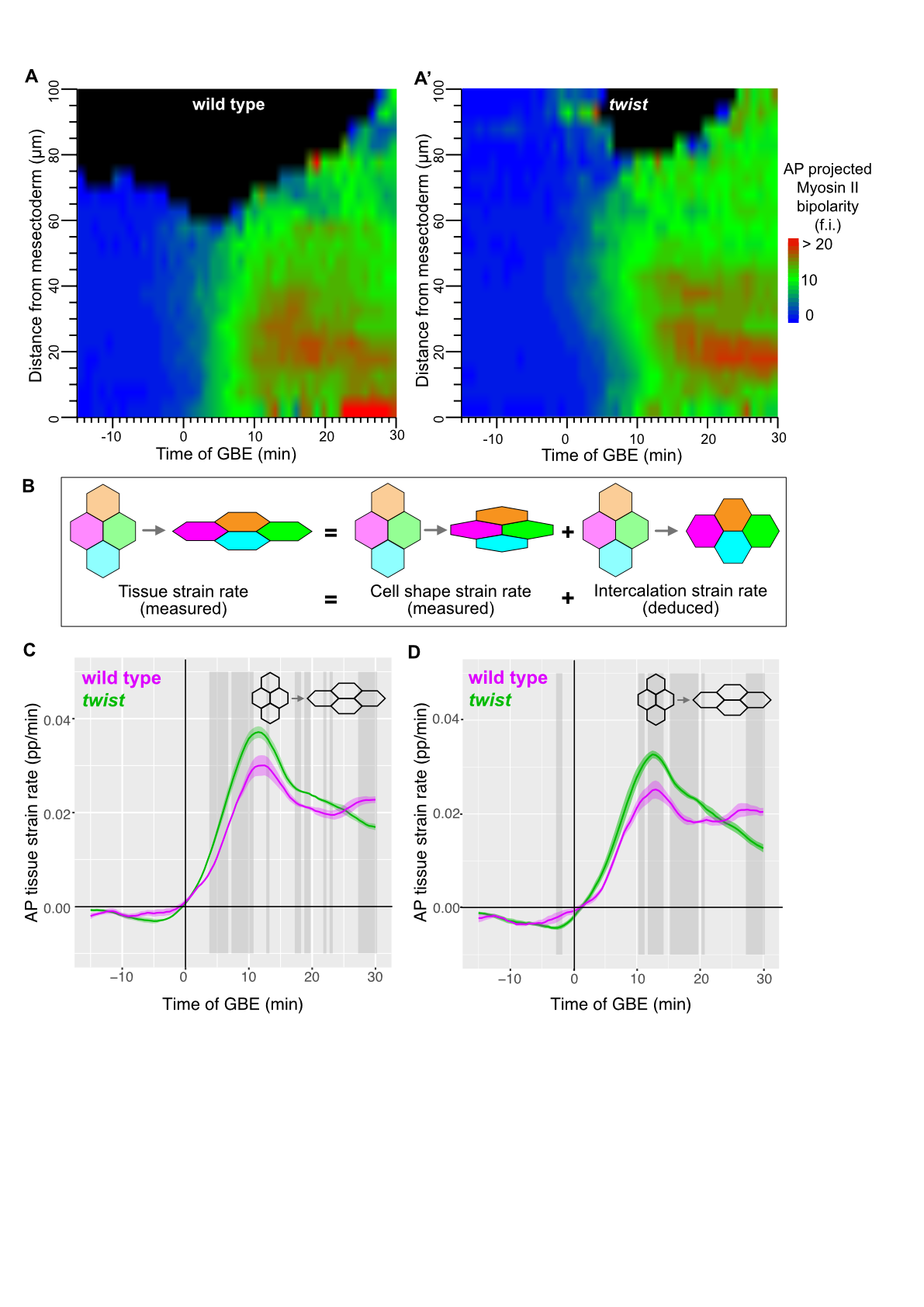

Supplement: S6 Fig — (A, A’) Myosin bipolarity projected along AP for all tracked ectodermal cells, summarised for wild type (A) and twist (A’) against time of GBE and distance from mesectoderm (μm) (see Methods). (B) Diagram illustrating that tissue strain rates are the sum of cell shape strain rates and cell intercalation strain rate [40]. Tissue and cell shape strain rates are measured and intercalation strain rates are deduced from those. (C) AP tissue strain rate plotted against time of GBE, summarising data from the full imaged region of 4 wild-type and 6 twist movies. (D) AP tissue strain rate plotted time of GBE summarising data from the 100-μm central region (see Methods) of wild-type (magenta) and twist (green) movies (see also S5 Fig). Data associated with this figure can be found in S9 Data. AP, antero-posterior; GBE, germband extension. (TIFF) [file pbio.3002611.s006.tiff]

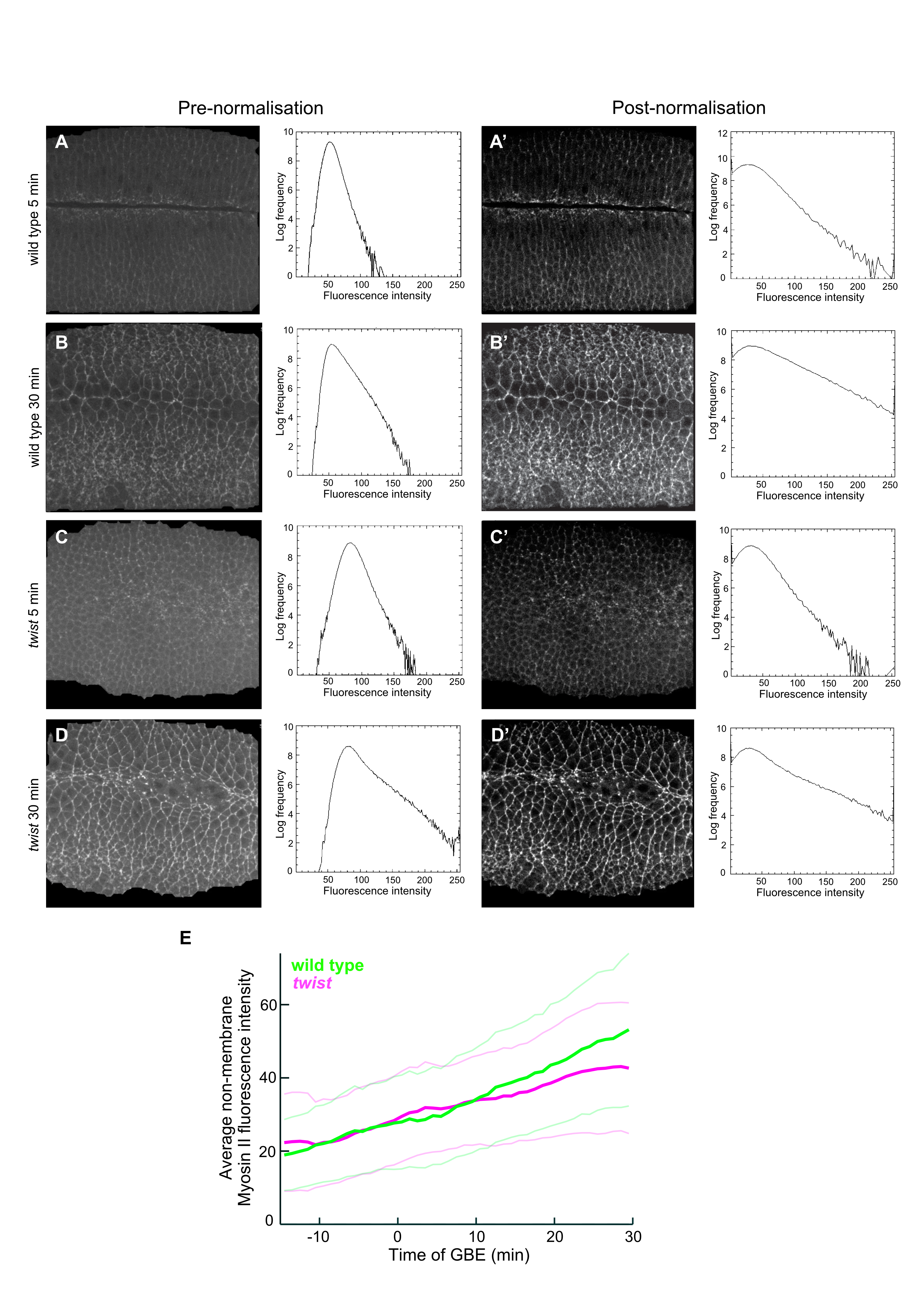

Supplement: S7 Fig — Images of the Myosin II channel from movies prior to (A-D) and after (A’-D’) normalisation, for example, wild-type (200910_3) (A-B’) and twist (201119_1) (C-D’) movies. The normalisation method used was designed to enable meaningful comparison of Myosin II intensities between movies (compare A’ to C’ and B’ to D’) while maintaining the observed differences in Myosin II intensity over time (compare 5-minute and 30-minute time points). See Methods for details. (E) Comparison of average “nonmembrane” apical Myosin II intensity in the ectoderm of 4 wild-type (magenta) and 6 twist (green) movies (see Methods). Fainter lines show standard deviations. Data associated with this figure can be found in S10 Data. (TIFF) [file pbio.3002611.s007.tiff]
