# Supplementary material for: Polarised cell intercalation during Drosophila axis extension is robust to an orthogonal pull by the invaginating mesoderm
Source: PLoS Biol. 2024 Apr 29;22(4):e3002611. doi: 10.1371/journal.pbio.3002611 (PMC11081494; doi:10.1371/journal.pbio.3002611)
Supplement: Attachment — Submitted filename: Response to reviewers_Lye et al.pdf [file pbio.3002611.s024.pdf]

Summary of changes:

We thank the reviewers for the quality and usefulness of their comments. In response, we have made the following changes:

- In response to Reviewer 1, we have included AP tissue strain rate graphs as a measure of germband extension (S6C,D Fig) and added a new section at the end of the Results describing these.
- In response to Reviewer 3, we have included more information about how we normalize our Myosin II quantifications, in the Methods and in a new S7 Fig.
- In response to Reviewer 4, we have added a new section in the Discussion with the heading "Comparison with other studies", on the discrepancy between findings in this work and previous studies, including our own (Butler et al, 2009).
- In response to Reviewer 4, we have more information on our statistics in the Methods, under "Statistics and graphical representations".
- In response to Reviewer 4, we have expressed junctional shrinkage and junctional growth in the same units.
- Throughout the manuscript, we have reviewed the text and figures for clarity, including revising our diagrams and updating our labels of axes in graphs.
- There are now 4 movies instead of 2 accompanying the manuscript
- We added new panels and reorganized supplementary figures as follow:
    - Fig 2: new panels G (quantification gradient of DV cell stretch) and I (diagram to explain "axial cell elongation" measure)
    - Fig 4: new panel A (change of units)
    - S4 Fig (combines previous figure 2-S1 and Figure 6-S1): new panel A (mesoderm invagination timings) and E (change of units)
    - S5 Fig (previously Figure 5-S1): new panels in B and C (plots for individual movies)
    - S6 Fig (previously Figure 6-S1): New panel in B (strain rates diagram), C and D (AP tissue strain rates)
    - S7 Fig: new figure to show examples of normalization for our Myosin II quantifications

The revised manuscript is accompanied by 7 Figures (Fig 1 to Fig 7), 7 supplementary figures (S1Fig to S7Fig) and 4 movies (Movie 1 to Movie 4).

Point-by-point response to reviewers:

Reviewer #1 (Evidence, reproducibility and clarity (Required)):

Summary: In this manuscript by the Sanson group, Lye and colleagues try to definitively answer the question of whether pulling forces from the ventral mesoderm have significant effects on convergent extension in the Drosophila germband (germband extension). While germband extension does occur in mutant embryos lacking mesoderm invagination, it has long been an open question in the field as to whether ventral pulling forces from the mesoderm have significant

effects (positive or negative) on cell intercalation during germband extension. To definitely address this question, Lye and colleagues generated high-quality, directly comparable datasets from wild-type and twist mutant embryos, and then systematically assessed nearly all aspects of cell intercalation, myosin recruitment, and tissue elongation over time. They demonstrate that pulling forces from the ventral mesoderm have negligible impacts on the course of germband extension. While there are indeed some interesting differences between wild-type and twist embryos with respect to cell intercalation and myosin recruitment, such differences are relatively minor. They conclude that the events of germband extension neither require nor are strongly affected by external forces from the mesoderm. While this is largely a negative results paper, I believe that it should be published and that it will be an impactful paper within the field. Namely, it will settle once and for all the question of whether mesoderm invagination is required for optimal germband extension in the early Drosophila embryo, and it suggests that tissues are largely autonomous developmental units that are buffered from outside mechanical inputs.

<u>Major comments:</u>

It seems to me that the one obvious omission from this paper is a general measure of convergent extension over time. I think it would be useful to the reader to include some measure of change in tissue aspect ratio over time between wild-type and twist embryos. This could be included in Figure 5 or 6.

We had already included graphs of tissue strain rates along AP (which measures tissue extension) for each of our movies in S3A,B Fig, because we use AP tissue strain rates to synchronize our movies within and between genotypes. But we agree it is a good idea to address this point more directly, so we have added two new panels comparing AP tissue strain rates in wild-type and *twist* embryos, in S6C,D Fig. Note that these are respectively for the whole of the imaged region (S6C Fig) and for a narrower, more central, AP region (S6D Fig). We have also added a new section at the end of the Results, under the heading “Comparing tissue extension rate in wild type and *twist* mutants”, to describe these results.

Note that in our measures, the “tissue” strain rate is decomposed into contributions from two cell behaviors, the “cell shape” strain rate and the “cell intercalation” strain rate (Blanchard et al., 2009; Butler et al., 2009), see new explanatory diagram in S6B Fig. “Tissue” and “cell shape” strain rate are directly measured, and “cell intercalation” strain rate is what remains when “cell shape” strain rate is removed from “tissue” strain rate. The “cell intercalation” strain rate calculated in that way is a “continuous” measure of cell intercalation, measuring the progressive shearing of cells during convergent extension. We also use a “discrete” measure of cell intercalation, which measures the number of cell neighbour exchanges, also called T1 transitions or swaps (we use the term T1 swaps throughout this manuscript). We found that both “continuous” and “discrete” measures of cell intercalation are unchanged in *twist* mutant compared to wild-type embryos (Fig 6F and 6E, respectively). In contrast, we find that the “cell shape” strain rate is increased in *twist* mutants (Fig 5B). Consistent with this finding, the “tissue” strain rate is also increased in *twist* mutants (S6C Fig).

Otherwise, I have no major comments on the experimental approach or the findings of this manuscript. It seems to me a straightforward and systematic approach for determining whether mesoderm invagination affects germband extension. I do have several minor comments that should be addressed prior to publication (below).

Minor comments:

I understand why cells would initially stretch more along the DV axis in wild-type embryos compared with twist embryos, but why do cells become so much more stretched along the AP axis (and become smaller apically) after 10 minutes of GBE in wild type compared with twist (Figure 2C and E). I think this is an interesting and non-intuitive result that would warrant a bit of explanation/conjecture.

This is not exactly what Fig 2C and E show, so we have worked on improving the clarity of the description by i) updating our schematics in Fig 2 (also for all the figures), and ii) changing the text in Results under the heading "The invaginating mesoderm pulls on the extending ectoderm" (lines 228 to 254). In Fig 2C for wild type, positive values show cells stretching along DV (with the rate of stretch increasing and then decreasing) and negative values show cells contracting along DV (with the rate of contraction increasing and then decreasing). In *twist* mutants, there is no DV stretching, but a little bit of DV contraction happens during GBE. The strong rate of DV contraction between 7.5 and 20 mins in wild type could in part be due to the endoderm invagination pulling in the orthogonal direction (AP) and helping the cells regaining a more isotropic shape. In Fig 2E, the rate of change in cell area follows a similar time course in the wild type, showing that the cells are increasing their areas until about 10 mins (positive values) and then reduce their areas again until the end of GBE (negative values). Note that the graph does not show raw (instantaneous) cell areas as suggested by the comment, but rather a rate of change (cell area strain rate).

So in wild type, the cells get stretched by the invaginating mesoderm, and once the mesoderm is not pulling anymore, the cells return to an isotropic shape. As there is no stretching in *twist* mutants, there is no equivalent restoration of the cell shapes along DV. Note that in *twist*, there is a milder increase in cell area in the first 15 mins of GBE (Fig 2E). This could again be caused by the pull from endoderm invagination stretching the cells along AP, which, as we have shown before, increases both cell shape strain rates along AP and cell areas (Butler et al., 2009). So the pull from endoderm invagination (along AP) will have an impact on cell area rates of change and possibly also, indirectly, on DV cell shape strain rates, in both *twist* and wild type embryos, during most of GBE. Therefore cell area and DV cell shape strain rates are affected by more than one process during GBE. In this paper, we investigate the impact of mesoderm invagination, which happens around the start of GBE, so have focused our analysis of the graphs in the Results to this period, and the differences between wild type and *twist.* However, to address the referee's question, we now comment on this in the discussion under the heading "Restoration of DV cell length after being stretched by mesoderm invagination".

To further improve clarity, we have mapped the timings of mesoderm invagination by eye in the movies (S4A Fig), which gave us an average time for both the start and end of mesoderm invagination relative to GBE onset (about -13 mins and +7 mins, respectively), which we now show on Fig 2C and Fig 2D graphs (yellow bar under x-axis).

We have also added a new panel to show more directly the gradient of cell elongation along DV in wild type compared to *twist* (Fig 2G). The graph shows the averaged DV cell shape strain rate from -7.5 to 7.5 minutes of GBE, for wild type and *twist* against distance from mesectoderm. This gives a quantification of the gradient that can be seen by eye in the spatiotemporal plot for the wild type in Fig 2D (red signal), which is absent in *twist* (Fig 2D').

We also added in Fig 2 a new panel with a diagram to explain more clearly our measure of "Axial shape elongation".

I don't understand how you are defining cell orientation in Figure 2G. How are you choosing the cell axis that you are then comparing with the body axis? Is it the long axis, or something more complicated than that? I think you should briefly provide this information in the results section. If it is included in the methods, I wasn't able to locate it.

Note this panel corresponds now to 2H in the revised manuscript. Yes, it is the orientation of the long axis of the cell relative to the antero-posterior embryonic axis. We have added a sentence in the Methods to clarify, line 918 "Cell orientation is the orientation of the long axis of the cell relative to the antero-posterior embryonic axis'. In the legend, we write "Average orientation <u>of principal axis</u> (degrees from AP axis) of ectodermal cells against time of time of GBE for wild type (magenta) and *twist* (green)."

Figure 2: Since you have the space, it might help the reader if you simply wrote out "strain rate" for panels B, D, and F, rather that used the abbreviation "SR."

Thank you for this suggestion, we have removed abbreviations in the labels throughout the manuscript and also reviewed these for clarity.

Please ensure that all axis labels are fully visible in the final figures. In several figures, the Y-axis labels were cut off (e.g., Fig 2I, 4A, 4D, 6B, 6C).

These were visible to us in our submitted version, but we will ensure everything is visible in the final version.

Where space permits, I would suggest using fewer abbreviations in axis labels to increase readability of the figures (e.g., in Figures 3H or 4D).

Now done.

In Figure 7, I would move the wild-type panels to the left and the twist panels to the right. I think it is more conventional to describe the normal wild-type scenarios first, and then contrast the mutant state.

Now done.

To be consistent with the literature, "wildtype" should be hyphenated (wild-type) when used as an adjective, or two separate words (wild type) when used as a noun.

Now done.

Reviewer #1 (Significance (Required)):

Advance: The advances in this manuscript are largely methodological, but the experiments and analyses are quite rigorous and allow the authors to make strong conclusions concerning their hypotheses. Their findings are based on a high-quality collection of movies from control and twist mutant embryos expressing a cell membrane marker and knock-in GFP-tagged myosin. Importantly, I think the researchers were correct in choosing to analyze twist single-mutant embryos (as opposed to snail or twist, snail double-mutant embryos), as the overall embryo geometry of these mutants is fairly similar to wild-type embryos, allowing the researchers to directly compare cell behaviors and myosin dynamics during germband extension. This approach also allows them to avoid indirect effects on the germband due to a completely non-internalized mesoderm.

Audience: The primary audience for this article will be basic science researchers working in the early Drosophila embryo who are interested in the interplay between the germband and neighboring tissues. Secondary audiences will include developmental biologists more broadly who are interested in biomechanical coupling (or in this case decoupling) of neighboring tissues.

Describe your expertise: I have been a Drosophila developmental geneticist for over twenty years, and I have been working directly on Drosophila germband extension for over a decade. I have published numerous papers and reviews in this field, and I am very familiar with the genetic backgrounds and types of experimental analyses used in this manuscript. Therefore, I believe I am highly qualified to serve as a reviewer for this manuscript.

Reviewer #2 (Evidence, reproducibility and clarity (Required)):

In the present manuscript, Lye et al. describe a highly detailed quantification of cell shape changes during germband extension in Drosophila melanogaster early embryo. During this process, ectodermal tissue contracts along the dorso-ventral axis, simultaneously expanding along the perpendicular antero-posterior direction, migrating from the ventral to the dorsal surface of the embryo as it extends. This important morphogenetic event is preceded by ventral furrow formation when mesodermal tissue (located in the ventral part of the embryo) contracts along the dorso-ventral axis and invaginates into the embryonic interior. The study compares cell shape dynamics in the wildtype Drosophila with that in the twist mutant, which largely lacks mesoderm and does not form ventral furrow. The major motivation of the study is to examine whether cellular behaviors and myosin recruitment in the ectoderm is cell autonomous, or if those cellular behaviors depend on mechanical interactions between mesoderm and ectoderm.

The authors first examine whether transcriptional patterning of key genes involved in germband extension is different between the wildtype and the twist mutant and find no significant difference. Next, the authors thoroughly quantify cellular behaviors and patterns of myosin recruitment in the two genetic backgrounds. A number of different measures are investigated, notably the rate of change in the degree of cellular asymmetry, rate of cell area change, rate of change of cell orientation, differences in myosin recruitment to cell edges of various orientation, as well as the rates of growth, shrinkage, and re-orientation of the various cellular interfaces. It is thoroughly documented how these quantities change as a function of developmental timing and spatial position within the embryo. These data serve basis for quantitative comparison between cellular dynamics in the two genetic backgrounds considered.

Overall, the study shows that cellular behaviors observed in the ectoderm are largely the same during the period of time following ventral furrow formation, as would be expected if those cellular behaviors were predominantly cell autonomous and not dependent on stresses generated in the mesoderm.

The data presented in the manuscript are of excellent quality and presentation is very clear.

Minor comments: none

Reviewer #2 (Significance (Required)):

I find that the study provides a thorough quantification of cell behaviors in a widely studied important model of morphogenesis. The work may be of particular interest for future model-to-data comparison, perhaps providing a basis for future modeling work. I therefore certainly think that this work warrants publication.

However, the results of the study largely parallel previous findings and do not appear novel or surprising. It is well established that in snail mutant that lack mesoderm entirely, germband extension proceeds largely normally. This well-established fact suggests that since tissue

dynamics in complete absence of mesoderm are largely unaffected, behaviors of individual cells are likely to not be affected either.

The work is pretty much entirely observational, and for most part provides a more detailed documentation/quantification of previous findings. I do not think it is appropriate for high profile publication.

We are not sure which evidence the reviewer is referring to here specifically. We agree that the single mutants *twist* or *snail*, or the double *twist snail* mutants do extend their germ-band. However, the question we are asking here, is *how well* do they extend their germband and to answer this question, quantitation is needed. The first quantitation of GBE were performed by (Irvine and Wieschaus, 1994). While they quantified GBE in various mutant contexts, they did not perform quantitation for *snail*, *twist,* or *twist snail* mutants. Instead, they refer to these mutants once in p839, with the following sentence: *"Additionally, twist and snail mutant embryos, which lack mesoderm, extend their germbands almost normally (Leptin and Grunewald, 1990; Simpson, 1983)."*

Following these earlier qualitative observations, various studies have quantified different aspects of GBE in mesoderm invagination mutants, with contradictory results. For example, some studies, including from our own lab, report a reduction in cell intercalation in the absence of mesoderm invagination (Butler et al., 2009; Wang et al., 2020), but there have also been reports that tissue extension and T1 transitions occur normally (Farrell et al., 2017)(see also the Introduction of our manuscript). These contradictory results have motivated our present study, and we have implemented rigorous comparison between wild type and mesoderm invagination mutants, being careful i) to check that the regions analyzed were comparable in terms of cell fate, and ii) to control for any confounding effects between experiments (see also response to reviewer 4, main question 2). We have also considered which mesoderm invagination mutants to use. We rejected *snail* or *twist snail* mutants because the absence of *snail* means that the mesodermal cells do not contract and thus stay at the surface of the embryo, which changes the spatial configuration of the embryo considerably and would make a fair quantitative comparison very difficult. Instead, we decided to use *twist* mutants, as in those, cell contractions still happen so the cells do not take as much space at the surface of the embryo, but the contractions are uncoordinated which means that there is no invagination (and we demonstrate here, no significant pulling on the ectoderm). We note that reviewer 1 highlights the merit of settling the question of the impact of mesoderm invagination on GBE and the pertinence of choosing *twist* mutants versus the alternatives (see also response to reviewer 4, suggestion 1).

Reviewer #3 (Evidence, reproducibility and clarity (Required)):

During morphogenesis, the final shape of the tissue is not only dictated by mechanical forces generated within the tissue but can also be impacted by mechanical contributions from surrounding tissues. The way and extent to which tissue deformation is influenced by tissue-

extrinsic forces are not well understood. In this work, Lye et al. investigated the potential influence of Drosophila mesoderm invagination on germband extension (GBE), an epithelial convergent extension process occurring during gastrulation. Drosophila GBE is genetically controlled by the AP patterning system, which determines planar polarized enrichment of non-muscle myosin II along the DV-oriented adherens junctions. Myosin contractions drive shrinking of DV-oriented junctions into 4-way vertices, followed by formation of new, AP-oriented junctions. This process results in cell intercalation, which causes tissue convergence along the DV-axis and extension along the AP-axis. In addition, GBE is facilitated by tissue-extrinsic pulling forces produced by invagination of the posterior endoderm. Interestingly, some recent studies suggest that the invagination of the mesoderm, which occurs immediately prior to GBE, also facilitates GBE. In the proposed mechanism, invaginating mesoderm pulls on the germband tissue along the DV-axis; the resulting strain of the germband cells generates a mechanotransduction effect that promotes myosin II recruitment to the DV-oriented junctions, thereby facilitating cell intercalation. Here, the authors revisited this proposed mechanotransduction effect using quantitative live imaging approaches. By comparing the wildtype embryos with twist mutants that fail to undergo mesoderm invagination, the authors show that although the DV-oriented strain of the germband cells was greatly reduced in the absence of mesoderm pulling, this defect had a negligible impact on junctional myosin density, myosin planar polarity, the rate of junction shrinkage or the rate of cell intercalation during GBE. A mild increase in the rate of new junction extension and a slight defect in cell orientation were observed in twist mutants, but these differences did not cause obvious defects in cell intercalation. The authors conclude that myosin II-mediated cell intercalation during GBE is robust to the extrinsic mechanical forces generated by mesoderm pulling.

Overall, I found that the results described here are very interesting and of high quality. The data acquisition and analyses were elegantly performed, statistics were appropriately used, and the manuscript was clearly written. However, there are a few points where some further explanation or clarification is necessary, as detailed below:

1. The main conclusion of the manuscript relies on appropriate quantification of myosin intensity at cell junctions. It is therefore important that the methods of quantification are well justified. Below are a few questions regarding the methods used in the analyses:

Thank you for this feedback. In response, we now provide a more detailed description of the normalization we use, in a new section of the Methods under the heading “Normalisation of Myosin channel” (line 985 onwards). We also provide a new supplementary figure (S7 Fig) to show examples of before and after normalization images at different timepoints.

-For myosin quantification, the authors state that "Background signal was subtracted by setting pixels of intensities up to 5 percentile set to zero for each timepoint" [Line826]. The rationale for selecting 5 percentile as the threshold for background should be explained. Also, how does this background value change over time?

For our normalization method, we stretched the intensity histogram of images to use the full dynamic range for quantification and enable meaningful comparison of intensities between different movies. The 5th percentile was chosen to set to zero intensity as this removed background signal without removing any structured Myosin signal (i.e., non-uniform, low level fluorescence - this was assessed by eye) (See Normalisation of Myosin channel in Methods).

Since the cytoplasmic signal is uniform, it is difficult to discern from true 'background', therefore some cytoplasmic signal might be set to zero with this method, but all medial and junctional Myosin structures will still be visible and have none-zero intensity values. However, since cytoplasm takes up a large majority of pixels in the image, and we only set 5% of pixels to zero, the majority of the cytoplasm will have non-zero pixel values. 'Background' increases slightly as Myosin II levels increase in general over time during development.

-*The authors mention that "Intensities varied slightly between experiments due to differences in laser intensity and therefore histograms of pixel intensities were stretched" [Line828]. The method of intensity justification should be justified. For example, does this normalization result in similar cytoplasmic myosin intensity between control and twist mutant embryos?*

As stated above, we stretched the intensity histogram of images to enable meaningful comparison of intensities between different movies, as stretching the histograms would bring Myosin II structures of similar intensities into the same pixel value range. We chose to stretch histograms using a reference timepoint (30 minutes, the latest timepoint analyzed), rather than on a per timepoint basis, because we saw a general increase in Myosin II over time, and we wanted to ensure that this increase was preserved in our analysis. This can be seen now in the examples shown in S7 Fig.

Note that we quantify Myosin II from 2 μm above to 2 μm below the level of the adherens junctions (see Methods), not throughout the entire cell, and therefore we have no true measure of cytoplasmic Myosin II. However, we can plot non-membrane Myosin II from this same apicobasal position in the cell. Non-membrane Myosin will include both the cytoplasmic signal and the Myosin II medial web (see above). When plotting these (new S7E Fig), we find that Myosin II intensities in this pool are similar in wild type and *twist*, confirming that that we are not inappropriately brightening one set of images compared to the other (e.g., *twist* versus wild type).

Finally, our measures of rate of junction shrinkage and intercalation are consistent with our Myosin II quantification results (see Figures 4A, D and 6E, F). This further validates our methods.

- A previous study demonstrates that the accumulation of junctional myosin is substantially reduced in twist mutant embryos compared to the wild type (Gustafson et al., 2022). In that work, junctional myosin was quantified as (I_junction - I_cytoplasm)/I_cytoplasm. In contrast, the cytoplasmic myosin intensity does not appear to be subtracted from the quantification in this study. How much of the difference in the conclusions of the two studies can be explained by this difference in myosin quantification?

As explained above, we choose to normalize our data by stretching histograms, rather than subtracting and dividing intensities between different pools of Myosin. The setting pixels of intensities up to 5 percentiles set to zero for each will have a similar effect to subtracting a small fraction of the cytoplasmic pool. We note that the intensity measurements in (Gustafson et al., 2022) are in the apical-top 5µm of the cell, and therefore their 'cytoplasmic' signal is likely to also include the apical medial web of Myosin. Also, after subtraction they use division by the cytoplasmic intensity in an attempt to bring pixel intensities between different movies into a comparable range, whereas we do this by stretching the histograms themselves (see above). We carefully designed our method to preserve the increase in Myosin II levels that we see over time in our post-normalization data. This is something that their method of normalization would not be predicted to capture, if their 'cytoplasmic' signal increase over time as well as their junctional signal. Indeed, in FigS6D of their paper, Myosin II levels do not appear to increase over time in these (presumably normalized) images.

Additionally, we note that in (Gustafson et al., 2022), not all Myosin II is fluorescently tagged since they use a *sqhGFP* transgene located on the balancer chromosome. This means that the line they use will have a pool of exogeneous Myosin tagged with GFP (expressed from the *CyO* balancer) and a pool of endogenous Myosin (expressed from the *sqh* gene on the X chromosome. It is not known whether endogenous and exogeneous GFP-tagged Myosin II will be recruited equally to cell junctions when in competition with each other. Therefore, in their genetic background, the ratio of junctional/cytoplasmic *sqhGFP* might not reflect the true ratio. To avoid this potential caveat, in our study we have used a new knock-in of the Myosin II Regulatory Light Chain (MRLC), which tags the *sqh* gene at the endogenous locus (Proag et al., 2019). The line is homozygous viable and thus all the molecules of Myosin II Regulatory Light Chain (encoded by *sqh*), and thus the Myosin II mini-filaments, are labelled with GFP.

Additionally, we note that when comparing their images of Myosin II in wildtype and *twist* (Figure 5D and D' in Gustafson et al., 2022), the overall Myosin signal appears reduced in *twist* mutants (including in the head and posterior midgut, which is outside the area that they are claiming Myosin II is recruited in response to mesoderm invagination). This suggests that Myosin II is generally reduced in their *twist* mutants (or images thereof), which is not expected and might indicate issues with their methods.

Therefore, differences in the methods may explain the discrepancies between studies. Importantly, we have quantified junctional shrinkage rates and intercalation, and our analysis of these rates is consistent with our Myosin II quantification results (see above).

-The authors used the tissue flow data to register the myosin channel and the membrane channel, which were acquired at slightly different times. The accuracy of this channel registration should be demonstrated.

As stated in our Methods, line 951 onwards: "the channel registration was corrected post-acquisition in order that information on the position of interfaces in the Gap43 channel could be

used to locate them in the Myosin channel. Therefore the local flow of cell centroids between successive pairs of time frames in the Gap43 channel is used to give each interface/vertex pixel a predicted flow between frames. A fraction of this flow is applied, equal to the Myosin II to Gap43 channel time offset, divided by the frame interval. Because cells deform as well as flow, the focal cell's cell shape strain rate is also applied, in the same fractional manner as above."

The images in Fig 3B' and 3C' show the Myosin II, with quantified membrane Myosin superimposed on the image as a color-code. Images in Fig 3B and 3C show the (normalized) Myosin II. Comparison of these images demonstrates that the channel registration is accurate. We have added a reference to these images in the methods (lines 959 to 961).

2. The authors show that cell intercalation is not influenced in twist mutant embryos. However, a previous study demonstrates that the speed of GBE is substantially reduced in twist mutants (Gustafson et al., 2022). It would be interesting to see whether a similar reduction in the speed of GBE was observed in this study.

We do not see a reduction in the speed of GBE as reported by (Gustafson et al., 2022), but rather an increase (see response to Reviewer 1). This is demonstrated by the new "AP tissue strain rate" graphs (S6C, D Fig), described in the new final section of the Results. The increase in the "AP tissue strain rate" is because there is an increase in the "AP cell shape strain rate" contributing to extension (while "AP cell intercalation strain rate" is unchanged) (see response to Reviewer 1).

3. It has been previously shown that contractions of medioapical myosin in germband cells also contribute to cell intercalation. The authors should explain why medioapical myosin was not included in the comparison between wildtype and twist mutant embryos.

Indeed, it has been shown that there is a flow of medial Myosin towards the junctions (Rauzi et al., 2010). However, and as described in that paper, this flow 'feeds' the enrichment of Myosin II at shrinking junctions, and thus the junctional Myosin II can be taken as a readout of polarized Myosin II behavior. Additionally, medial flows are more technically challenging to quantify, especially when quantification is required in a large number of cells as is the case for our study.

Importantly, our junctional Myosin II and junctional shrinkage rate results are consistent with each other, therefore it is very unlikely that analyzing medial Myosin II would lead us to form a different conclusion. We have added a sentence in the Methods to explain why we chose to quantify junctional, and not medial, Myosin II (line 939 onwards).

Minor points:

1. Fig. 1-S1 panel C: the number of cyan cells changes non-monotonically. It first decreases from -10 min to 10 min, then increases from 10 min to 20 min. This is confusing since in theory the

number of tracked cells should not increase over time if the cells are tracked from the beginning of the movie.

(Now S1C Fig) the cyan cells highlight tracked mesodermal and mesectodermal cells, which are not included in the analysis. The low number of mesodermal cells highlighted at 10mins germband extension is because mesodermal and mesectodermal cells are not always tracked successfully at this time, as they are very stretched and invaginating (changing plane). Note that the legend includes a note that *'"Unmarked cells are poorly tracked and excluded from the analysis"*. Also see Methods: *"Note on number of cells in movies, for notes on changes to the number of tracked ectodermal cells throughout the timecourse of the movies."*

2. Fig. 1-S2: the vnd band in panel A appears to be much narrower than in panel B.

(Now S2 Fig) These are fixed embryos, therefore this could be (at least partially) due to slight differences in exact developmental age of the embryo. Note that we wanted to check that *vnd* and *ind* are expressed in the correct places in the ectoderm. We were motivated to check this because the width of mesoderm is reduced in *twist*, so we thought it was important to verify that there is not a population of 'ectodermal' cells with a strange fate (i.e., negative for both *vnd* and i*nd*). Our experiments show that *vnd* abuts the mesoderm/mesectoderm in *twist* as in wildtype, and that the cells immediately lateral to the *vnd* cell population express *ind* as expected.

It is possible that there is a slight difference in the number of *vnd* cells in *twist* mutants compared to wildtype, but we see no differences in Myosin II bipolarity that would coincide with the *vnd/ind* boundary in the spatiotemporal plots (S4D, D' Fig). Therefore, this would not change the interpretation of our results. Counting the number of rows of *vnd* cells prior to any cell intercalation (the number of rows will reduce as cells intercalate) would be technically challenging as the lateral border of *vnd* expression is hard to discern at this time due to lower levels of *vnd* expression laterally within the *vnd* expression domain.

3. The schematic in Fig. 2J suggests that at the onset of mesoderm pulling the germband cells have a uniform angle of rotation (towards bottom right). Is this the case?

No, this schematic is purely supposed to show that as cells stretch, they also reorient. In response, we added the sentence in the legend "note that angle of rotation shown is arbitrary"

4. The description of myosin intensity normalization in the Methods section is somewhat difficult to follow [Line 829 - 832]. It would be helpful if the authors can show one or two images before and after intensity normalization as examples.

In response, we have now added some examples of before and after normalization images in a new supplementary figure (S7 Fig). We have also reviewed the Methods to improve clarity.

5. Line 704: "Z-stacks for each channel were collected sequentially" - the step size in Z-axis should be reported.

Thank you for this, the step size was 1μm. We have now added this information in the Methods (line 814).

6. Fig. 4C: what are the thin, black lines in the image?

This image is a 2D representation of the Gap43Cherry signal at the level of the adherens junctions extracted for tracking, not a simple confocal z-slice. When viewing these representations, you can see lines showing borders between where information from different z-stacks was used for the tracking layer. Unfortunately, our software does not allow us to remove these lines, but they do not affect tracking or quantification.

Reviewer #3 (Significance (Required)):

While most previous work on tissue mechanics and morphogenesis focuses on tissue-intrinsic mechanical input, recent studies have started to emphasize the contribution of tissue-extrinsic forces. An important challenge in understanding the function of tissue-extrinsic forces lies in the difficulties in properly comparing the wild type and the mutant samples that disrupt extrinsic forces, in particular when cell fate specification is altered in the mutants. In this work, the authors addressed this challenge by employing a number of approaches to warrant a parallel comparison between genotypes, including examining the AP- and DV-patterning of the tissue, selecting sample regions with comparable cell fate for analysis, and carefully aligning the stage of the movies. With these approaches, the authors provide compelling evidence to support their main conclusions. By teasing apart the role of the intrinsic genetic program and the extrinsic tissue forces, the work provides important clarifications on the function of mesoderm pulling in GBE and adds new insights into this well-studied tissue morphogenetic process. This work should be of interest to the broad audience of epithelial morphogenesis, tissue mechanics and myosin mechanobiology.

Reviewer #4 (Evidence, reproducibility and clarity (Required)):

Lye and colleagues investigate the impact of tissue-tissue interactions on morphogenesis. Specifically, they ask how disrupting mesoderm internalization affects convergence and extension of the ectoderm (germband) in Drosophila embryos. Using twi mutants in which mesoderm invagination fails, the authors find that the invagination of the mesoderm deforms germband cells, but does not significantly contribute to patterning, cell alignment, myosin polarization and cell-cell contact disassembly (which drive germband convergence). The authors find modest effects of mesoderm invagination on new junction formation and orientation (which drive extension), but

these changes do not have a significant effect on germband elongation. The authors conclude that germband extension is robust to external forces from the invagination of the mesoderm.

MAIN
1. The authors clearly show that myosin density is not different in wild-type and twi mutant embryos, and subsequently argue that the pulling force from the mesoderm does not elicit a mechanosensitive response in early germband extension. But if the cell density is constant, doesn't that mean that the longer, DV-oriented interfaces in the wild type accumulate more total myosin than their shorter counterparts in twi mutants? Assuming that the total number of myosin molecules per cell is not greater in the wild type, wouldn't increased total myosin at the membrane suggest a response to the increased deformation? Certainly the cells are able to maintain the same cell density despite the pulling force from the mesoderm, so can the authors rule out a mechanosensing mechanism?

To carify, we do not rule out a mechanosensing mechanism. We agree the total Myosin II at stretched interfaces is higher than at unstretched interfaces and propose a homeostatic mechanism to maintain Myosin II density on the cortex upon rapid stretching (summarized in Fig 7). Indeed it is possible that this mechanism could itself be due to mechanosensitive recruitment of Myosin II (though there are also other possibilities). This is discussed under the headings "Mechanisms regulating Myosin II density at the cortex and consequences for polarized cell intercalation" and "Restoration of DV cell length after being stretched by mesoderm invagination", in the Discussion. In response, we have worked on the clarity of these sections.

What happens to the Gap43mCherry signal? From Figure 2A, it seem to be diluted ventrally in the wild type as compared to twi mutants? Comparing myosin and Gap43 dynamics may shed light on whether myosin accumulates more or less than one would expect simply on the basis of having longer contacts.

We quantify the density of Myosin, rather than the total amount. Therefore, the length of the cell-cell contact should not matter. The suggestion of comparing Myosin density to Gap43Cherry density is in principle a good one, as it would allow us to compare a protein which is not diluted as cell contact length increases (Myosin) to one which appears to be (Gap43). However, it is not essential for the conclusions that we make. Also, in practice quantifying the Gap43Cherry signal would not be straightforward on our existing movies due to the imaging parameters used. We capture the Gap43Cherry channel (but not the Myosin channel) with a 'spot noise reducer' tuned on in the camera software, due to very occasional bright spot noise, which confuses the tracking software. Therefore, our Gap43Cherry signal is manipulated during acquisition and to quantify from these images would not be appropriate. Therefore, we would have to acquire, track and quantify some new movies, which is not possible within the timeframe of a revision.

In summary, we think that we have sufficient evidence from our analysis that Myosin II is not diluted upon junctional stretching without comparing to quantification of Gap43Cherry, and

the time investment required to quantify the Gap43Cherry would not be worthwhile as it would require more data to be acquired and processed.

2. The authors previously argued that mesoderm invagination was required for the fast phase of cell intercalation [Butler et al., 2009]. However, here the authors interpret that loss of twi does not significantly slow down interface contraction, but accelerates the elongation of junctions and cells along the AP axis, which overall would mean that mesoderm invagination is (slightly) detrimental for axis elongation. The discrepancy between their previous and current results should be discussed.

We agree that this is important to discuss and have now included this under a new heading "Comparison with other studies" in the Discussion. In a nutshell, we think that these discrepancies arise from the challenges of comparing wild-type and *twist* mutant embryos relative to each other, and as a consequence we have made various improvements to our methods since (Butler et al., 2009). These improvements included using markers that would be expressed at the same levels in wild-type and *twist* embryos. Additionally, we did not use overexpressed cadherin-FPs (namely, the *ubi-CadGFP* transgene), which may have confounding effects, and we used a knock-in *sqhGFP* to ensure we could all Myosin II molecules were labelled by GFP. We also carefully controlled the temperature at which we acquired the movies, standardized the level at which to track cells and quantify Myosin between movies, as well as improving the accuracy of our image segmentation and cell type identification since our previous study (Butler et al., 2009). See also response to reviewer 2.

3. Related to the previous point, it is surprising that the differences shown in Figure 4A-B are not significant. This is particularly troubling when in Figure 5B the authors claim a significant difference in cell elongation rate, which is higher in twi mutants (but only in very short time intervals and actually switches sign at the end of germband extension). These are just two examples, but I think the analysis of significance on a per-time point basis is problematic.
Have the authors considered analyzing their results as time series rather than comparing individual time points? Or perhaps integrating the different metrics over the duration of germband extension (e.g. using areas under the curve)? That way they would not have to arbitrarily decide if significant differences in a few time points should or not be interpreted as significant overall differences.

For graphs plotted against time of germband extension, we do not think it is appropriate to analyze as a time series rather than comparing individual time points, since different developmental events (such as mesoderm invagination) occur at different times. For graphs plotted against time to/from cell neighbor swap, these can also change over time (e.g., centroid-centroid orientation, Fig 6D). Therefore, we do not feel that it appropriate to run statistical analyses as a timeseries for these comparisons either. Statistically cut-offs are by their nature arbitrary. We have tried to mention non-significant trends throughout the text (including for Fig 4A, B), to highlight where there may be minor (but not significant) differences. We have reviewed

the text and made some changes throughout to clarify our interpretations of statistical significance and trends in the data.

4. While the number of cells analyzed is impressive, the number of embryos is relatively low, particularly for the wild type (only four embryos analyzed). If I understood correctly (if not, please clarify) the authors ran their statistics using cells and not embryos as their measurement unit. But I could not find any evidence that cells from the same embryo can be considered as independent measurements. This could be easily done by demonstrating that the variance of any of the measurements (e.g. elongation, area change rate, etc.) for cells in an embryo is comparable to that calculated when mixing cells from different embryos.

We do not simply use the number of cells as an n for our experiments. We use a mixed effects model for our statistics as previously (Butler et al., 2009; Finegan et al., 2019; Lye et al., 2015; Sharrock et al., 2022; Tetley et al., 2016). This estimates the *P* value associated with a fixed effect of differences between genotypes, allowing for random effects contributed by differences between embryos within a given genotype. We have added a sentence in the Methods under "Statistics and graphical representations" to make this clear.

MINOR
1. Figure 4D: the authors show no difference in the proportion of neighbor swaps per minute between wild-type and twi- mutant embryos. But how about the absolute number of neighbour swaps per minute? Does that change in twi mutants (and if so, why?).

The number of interfaces involved in a T1 swap are expressed as a proportion of the total number of DV-oriented interfaces for all tracked ectodermal germband cells, to take account of differences in the number of tracked cells between different timepoints and different movies. Presenting the absolute number of T1 swaps per minute could lead to misleading interpretations.

2. I was a bit confused about the reason why in Figure 4A the authors measure the rate of interface contraction in units of "proportion/min", but in Figure 5A they measure interface elongation in units of "um/min". Unless there is a good reason not to, these two metrics should be reported using the same units. Is there a difference in the rate of interface contraction when measured in absolute units (um/min)?

We thank the reviewer for this comment, which has prompted us to improve the clarity of our discussion of differences between junction shrinkage and growth between wildtype and *twist.* We now express both interface contraction and interface growth in units of proportion/minute (Fig 4A and Fig 5A). Interestingly, the period of significance is slightly shorter for junction growth when expressed as proportion/min compared to μm/min (Fig 5A), with only the *initial* growth rate being significantly higher in *twist* compared to wildtype, suggesting that it is the initial rate of junction growth is affected by the global force balance of boundary conditions (See lines 733 onwards in Discussion). Note that we also show interface contraction in units of μm/min in supplementary figure S4E Fig to highlight that yes, there is a difference in contraction rate when measured in

absolute units (μm/min). This is now discussed in Results and also in the Discussion.

3. The discussion of previous work on cell deformation within the mesoderm (page 16, first paragraph) should probably include recent work from Adam Martin's lab (e.g. [Heer et al., 2017]; or [Denk-Lobnig et al., 2021]).

Thank you, we have now added these references to the manuscript.

SUGGESTIONS

1. While I appreciate the arguments that the authors provide to use twi mutants rather than sna mutants or twi sna double mutants, as the authors indicate, in twi mutants there is still contractility in the mesoderm (albeit not ratcheted). Therefore, it is possible that contractile pulses from the mesoderm in twi mutants could still facilitate cell alignment and polarization of myosin in the germband. Given the previous results from the Zallen lab using twi sna double mutants (see above) this is unlikely to be the case, but the findings in this manuscript would be significantly stronger if they included similar analysis in the double mutants.

We had concerns about using *sna* or *twi sna* double mutants due to the large amount of space the un-internalized mesoderm takes up on the exterior of the embryo. This concern is also shared by reviewer 1 *"Importantly, I think the researchers were correct in choosing to analyze twist single-mutant embryos (as opposed to snail or twist, snail double-mutant embryos), as the overall embryo geometry of these mutants is fairly similar to wild-type embryos, allowing the researchers to directly compare cell behaviors and myosin dynamics during germband extension. This approach also allows them to avoid indirect effects on the germband due to a completely non-internalized mesoderm."* In addition to this concern, imaging of *snail* or *twist snail* embryos by confocal imaging to include the ventral midline (which is required to define embryonic axes) is problematic as the un-constricted mesodermal cells occupy virtually all the field of view, leaving very few ectodermal cells to analyze.

Whilst we acknowledge that there are some (un-ratcheted) contractions of mesodermal cells in *twist* mutants, we have clearly shown that there is no DV stretch and very little reorientation of cells (Fig 2). We have added a quantification of the gradient of DV stretch to make this point more clearly (Fig 2G). Therefore, any residual contractile activity in the mesodermal cells of *twist* mutants does not appear to have a mechanical impact on the ectoderm. We cannot exclude the possibility that there is some transmission of forces between contracting cells of the mesoderm and the ectoderm in *twist* mutants. However, our evidence suggests that the large tissue scale force that transmits to the ectoderm from the invaginating mesoderm is missing in *twist* mutants, and it was the effects of that force that we wished to investigate (See also response to reviewer 2).

Reviewer #4 (Significance (Required)):

This is an interesting study, with careful quantitative analysis of cellular and subcellular dynamics.

The results follow previous findings from Jennifer Zallen and the authors themselves. The Zallen lab showed that cell alignment, myosin polarization and germband extension are normal in sna twi mutants [Fernandez-Gonzalez et al., 2009], a result that the authors fail to cite. The results in the present manuscript are similar, but the analysis is much more in depth here, so the findings by Lye and colleagues certainly warrant publication.

In response to the referee's comment, we have added in the Discussion a citation of the Fernandez-Gonzalez (2009) paper mentioning that Myosin II cable-like structures appear normal later in germband extension in *snail twist* mutants. Note that we did not initially cite (Fernandez-Gonzalez et al., 2009) related to the point made by the referee, because the subject of their study is the formation of multicellular rosettes, not whether a pull from mesoderm affects Myosin II polarity and cell intercalation. The formation of multicellular rosettes occurs later in germband extension, and therefore these results are not directly comparable to the analyses that we present in our study. Additionally, their measures of alignment are defined as linkage to other approximately DV-oriented interfaces, rather than directly measuring orientation compared to the embryonic axes as we do here, as a different question is being addressed. Specifically, the quoted *snail twist* experiment is interpreted as extrinsic forces from the mesoderm not being required for linkage of Myosin enriched DV-oriented interfaces together. Myosin II quantification is more rudimentary with edges being assigned as Myosin positive or Myosin negative, as opposed to quantifying the density of Myosin on each interface; they also did not compare Myosin II quantification between wildtype and *twist* embryos.

However, there are a number of experimental points that I think need to be addressed to solidify the manuscript, particularly in terms of statistical analysis.

Please see more details above regarding specific concerns about experimental points and statistics (for example, main points 3 and 4). Additionally, we note that reviewer 3 states *"statistics were appropriately used"*, and our statistical methods are the same as we have used in previous studies comparing live imaging data (Butler et al., 2009; Finegan et al., 2019; Lye et al., 2015; Sharrock et al., 2022; Tetley et al., 2016).

REFERENCES

Blanchard, G. B., Kabla, A. J., Schultz, N. L., Butler, L. C., Sanson, B., Gorfinkiel, N., Mahadevan, L. and Adams, R. J. (2009). Tissue tectonics: morphogenetic strain rates, cell shape change and intercalation. *Nat Methods* **6**, 458-464.

Butler, L. C., Blanchard, G. B., Kabla, A. J., Lawrence, N. J., Welchman, D. P., Mahadevan, L., Adams, R. J. and Sanson, B. (2009). Cell shape changes indicate a role for extrinsic tensile forces in Drosophila germ-band extension. *Nat Cell Biol* **11**, 859-864.

Farrell, D. L., Weitz, O., Magnasco, M. O. and Zallen, J. A. (2017). SEGGA: a toolset for rapid automated analysis of epithelial cell polarity and dynamics. *Development* **144**, 1725-1734.

Fernandez-Gonzalez, R., Simoes Sde, M., Roper, J. C., Eaton, S. and Zallen, J. A. (2009). Myosin II dynamics are regulated by tension in intercalating cells. *Dev Cell* **17**, 736-743.

Finegan, T. M., Hervieux, N., Nestor-Bergmann, A., Fletcher, A. G., Blanchard, G. B. and Sanson, B. (2019). The tricellular vertex-specific adhesion molecule Sidekick facilitates polarised cell intercalation during Drosophila axis extension. *PLoS Biol* **17**, e3000522.

Gustafson, H. J., Claussen, N., De Renzis, S. and Streichan, S. J. (2022). Patterned mechanical feedback establishes a global myosin gradient. *Nat Commun* **13**, 7050.

Irvine, K. D. and Wieschaus, E. (1994). Cell intercalation during Drosophila germband extension and its regulation by pair-rule segmentation genes. *Development* **120**, 827-841.

Leptin, M. and Grunewald, B. (1990). Cell shape changes during gastrulation in Drosophila. *Development* **110**, 73-84.

Lye, C. M., Blanchard, G. B., Naylor, H. W., Muresan, L., Huisken, J., Adams, R. J. and Sanson, B. (2015). Mechanical Coupling between Endoderm Invagination and Axis Extension in Drosophila. *PLoS Biol* **13**, e1002292.

Proag, A., Monier, B. and Suzanne, M. (2019). Physical and functional cell-matrix uncoupling in a developing tissue under tension. *Development* **146**.

Rauzi, M., Lenne, P. F. and Lecuit, T. (2010). Planar polarized actomyosin contractile flows control epithelial junction remodelling. *Nature* **468**, 1110-1114.

Sharrock, T. E., Evans, J., Blanchard, G. B. and Sanson, B. (2022). Different temporal requirements for tartan and wingless in the formation of contractile interfaces at compartmental boundaries. *Development* **149**.

Simpson, P. (1983). Maternal-Zygotic Gene Interactions during Formation of the Dorsoventral Pattern in Drosophila Embryos. *Genetics* **105**, 615-632.

Tetley, R. J., Blanchard, G. B., Fletcher, A. G., Adams, R. J. and Sanson, B. (2016). Unipolar distributions of junctional Myosin II identify cell stripe boundaries that drive cell intercalation throughout Drosophila axis extension. *Elife* **5**.

Wang, X., Merkel, M., Sutter, L. B., Erdemci-Tandogan, G., Manning, M. L. and Kasza, K. E. (2020). Anisotropy links cell shapes to tissue flow during convergent extension. *Proc Natl Acad Sci U S A* **117**, 13541-13551.
